# Histone deacetylase (HDAC) inhibitors- based drugs are effective to control *Mycobacterium tuberculosis* infection and promote the sensibility for rifampicin in MDR strain

**DOI:** 10.1590/0074-02760230143

**Published:** 2023-12-22

**Authors:** Adrián Rodríguez-Carlos, Yolanda Jacobo-Delgado, Alan Orlando Santos-Mena, Mariana H García-Hernández, Luis Adrian De Jesus-Gonzalez, Edgar E Lara-Ramirez, Bruno Rivas-Santiago

**Affiliations:** 1 Medical Research Unit-Zacatecas, Mexican Institute for Social Security-IMSS, Zacatecas, Mexico; 2 Instituto Politécnico Nacional, Centro de Biotecnología Genómica, Laboratorio de Biotecnología Farmacéutica, Reynosa, Mexico

**Keywords:** drug repositioning, tuberculosis, innate immunity, histone deacetylase inhibitors, multidrug-resistant, Mycobacterium tuberculosis

## Abstract

**BACKGROUND:**

Tuberculosis (TB) is a major public health problem, which has been aggravated by the alarming growth of drug-resistant tuberculosis. Therefore, the development of a safer and more effective treatment is needed.

**OBJECTIVES:**

The aim of this work was repositioning and evaluate histone deacetylases (HDAC) inhibitors- based drugs with potential antimycobacterial activity.

**METHODS:**

Using an *in silico* pharmacological repositioning strategy, three molecules that bind to the catalytic site of histone deacetylase were selected. Pneumocytes type II and macrophages were infected with *Mycobacterium tuberculosis* and treated with pre-selected HDAC inhibitors (HDACi). Subsequently, the ability of each of these molecules to directly promote the elimination of *M. tuberculosis* was evaluated by colony-forming unit (CFU)/mL. We assessed the expression of antimicrobial peptides and respiratory burst using reverse transcription-quantitative polymerase chain reaction (RT-qPCR)

**FINDINGS:**

Aminoacetanilide (ACE), N-Boc-1,2-phenylenediamine (N-BOC), 1,3-Diphenylurea (DFU), reduce bacillary loads in macrophages and increase the production of β-defensin-2, LL-37, superoxide dismutase (SOD) 3 and inducible nitric oxide synthase (iNOS). While only the use of ACE in type II pneumocytes decreases the bacterial load through increasing LL-37 expression. Furthermore, the use of ACE and rifampicin inhibited the survival of intracellular multi-drug resistance *M. tuberculosis*.

**MAIN CONCLUSIONS:**

Our data support the usefulness of *in silico* approaches for drug repositioning to provide a potential adjunctive therapy for TB.

*Mycobacterium tuberculosis* (*Mtb*) is the main agent of tuberculosis (TB) in humans and is one of the leading causes of death worldwide from an infectious agent only after coronavirus disease 19 (COVID-19). Nowadays, 1.6 millions of people die due to TB, and approximately 10 million develop an active infection. Indeed, the World Health Organization (WHO) has estimated that at least one-fourth of the global population is latently infected with *Mtb*.^1^ Although conventional treatment for TB is effective, the resistance to antibiotics has increased exponentially in the last few decades.^2^ Thus, the WHO has implemented a strategy for the eradication of TB for 2035, which comprises the development of a safer and more effective treatment. One of the therapeutic options proposed is immunomodulation.^1^ ^,^ ^3^

The innate immune response against *Mtb* is a key factor determining the outcome of the infection. In recent years, several studies have reported that epigenetic modifications that regulate gene expression are determinants for the bacterial growth in macrophages and epithelial cells. The acetylation of DNA is the main epigenetic mechanism studied.^4^ This mechanism is regulated mainly by two enzymes: histone acetyltransferases (HAT), which adds acetyl groups to the histone, nuclear receptors, and transcription factors lysine. The histone deacetylases (HDAC) remove acetyl groups and promote gene repression, hereby, HDACs are valued as a therapeutic target for infectious diseases.^4^ There are 18 HDAC enzymes divided into four classes: Class I Rpd3-like proteins (HDAC 1-3 and HDAC 8); the Class II Hda1-like proteins (HDAC 4-7 and HDAC 9-10); the Class III Sir2-like proteins (SIRT 1-7); and the Class IV protein (HDAC 11).^5^

HDACs class I, II, and IV are zinc-dependent, thereby molecules that chelate zinc from the active enzymatic site are proposed as inhibitors. The therapeutic application of HDAC inhibitors (HDACi) to control *Mtb* has been valued by their immunoregulatory activity.^6^ For instance, the use of vorinostat, a non-selective HDACi, during *in vitro* infection of alveolar macrophages increased the production of IL-1β and reduced IL-10.^7^ Similarly, tubastatin A (TSA) up-regulates TNF-α, IL-12p40, and IFN-γ expression by inhibiting HDAC 6 in a mouse TB model.^8^ In addition, *Mtb* clearance under HDACi treatment has been associated with an improved innate immune response.^6^

Antimicrobial peptides (AMPs) are key effector molecules of the innate immune and they have been considered as a link between innate and adaptive immunity. AMPs have shown important bactericidal activity and a low rate of antibacterial resistance.^9^ Therefore, they have been considered as an alternative to conventional antibiotics. The most relevant AMPs that have shown *Mtb* killing activity are the cathelicidin LL-37 and the human β-defensin-2 (HBD-2).^10^ Indeed, these AMPs can be induced by both endogen and exogen molecules. For instance, Kai Zhang et al. reported that sodium butyrate induced the expression of LL-37 in *M. bovis* infected macrophages, promoting mycobacteria elimination.^11^ Interestingly, other studies have reported similar observations using entinostat, which potentiates the induction of HBDs and cathelicidin by 20-to-30-fold times more than sodium butyrate.^12^

Nowadays, entinostat is used for cancer treatment, nonetheless, its efficacy to induce AMPs expression makes it an excellent candidate for the treatment of *Mtb*. Furthermore, entinostat is known to regulate transcription factors, such as STAT3 which is involved in AMP expression.^13^ The presence of the functional aroilated group phenylenediamine in entinostat, is essential to induce AMPs expression.^6^ Despite the effectiveness reported for entinostat to promote the innate response, its cost represents a clinical hurdle. Hereby, the aim of this work was to search, repositioning and evaluate HDAC inhibitors- based drugs with potential antimycobacterial activity with a low cost.

## MATERIALS AND METHODS

*Ligand-based search and molecular docking* - The ZINC15 database (https://zinc15.docking.org) was used to search for structural molecules similar to phenylenediamine or entinostat, considering a Tanimoto index similarity value ≥ 0.60. The phenylenediamine or entinostat like molecules were then evaluated as capable of inhibit the HDAC1-3 isoforms using the SEA program (Similarity ensemble approach, http://sea.bkslab.org/). These results were also confirmed by molecular docking using the Autodock Vina software (http://vina.scripps.edu/).^14^ The reference value for the binding affinity was the prototype molecule (entinostat). The interaction between molecules and enzymes was measured, choosing only molecules with a binding energy (ΔG) ≤ -5.00 kcal/mol. In addition, were considered adverse effects, and the price of acquisition. After these bioinformatic analysis, three molecules out of two hundred were chosen: aminoacetanilide (ACE), N-Boc-1,2-phenylenediamine (N-Boc), and 1,3-Diphenylurea (DFU) were selected based in their low cost and lower adverse effects. All molecules were obtained commercially (Sigma-Aldrich St Louis MO, USA). The cytotoxicity of each compound was evaluated for each kind of cell used in the present study using the Guava ViaCount Assay (Luminex, Austin Tx, USA), which distinguishes between viable and non-viable cells based on the differential permeability of DNA-binding dyes.

*Bacterial culture* - *Mtb* (Strain H37Rv, ATCC 27294, Manassas, VA, USA) and the clinical isolated strain of multidrug resistant *Mtb* (MDR-MTB) were cultured in Middlebrook 7H9 medium (Becton Dickinson, Franklin Lakes, NJ. USA) supplemented with 0.2% glycerol, 0.5% Tween 80 and, 10% oleic acid, albumin, dextrose, and catalase (OADC enrichment medium; BBL, Becton Dickinson, Franklin Lakes, NJ, USA) and were incubated at 37ºC with 5% CO_2_ atmosphere. The strains were separated into working aliquots of 1.2 × 10^8^ colony-forming unit (CFU)/mL and kept frozen until use. Aliquots were centrifuged for 5 min at 6000×g, and the resulting *Mtb* pellets were declumped by vortexing (5 min) with five sterile 3-mm glass beads in 1 mL of RPMI medium enriched with 10% pooled human AB serum (Biowest, Nuaille, FR). The remaining *Mtb* clumps were removed with an additional centrifugation step at 350×g for 5 min suspension volumes required to obtain the desired multiplicity of infection (MOI 5) were calculated based on the CFU numbers known to be present in the *Mtb* supernatants. The actual CFU number used for *in vitro* infections were confirmed in each experiment by plating onto agar 7H10. *Mtb* concentrations of the frozen stock suspensions were confirmed by counting CFUs/mL on 7H10 agar plates using serial dilutions in triplicate.

*Evaluation direct antimicrobial activity* - The direct antimicrobial activity of ACE, N-Boc-1,2-phenylenediamine, and 1,3-DFU was assayed by the Resazurin Microtiter Assay Plate method. Resazurin, an oxidation-reduction indicator, has been used in antimicrobial assays in previous studies.^15^ The growth medium used for *Mtb* was 7H9 broth supplemented with 10% Middlebrook OADC (oleic acid, albumin, dextrose and catalase) (Becton-Dickinson, Sparks, MD, USA). A 10% (w/v) resazurin solution (sodium salt, Sigma, St. Louis, MO, USA) sterilised by 0.22 µM ﬁltration was used. Serial dilutions were evaluated to six final concentrations of 3.90 to 250 μg/mL (treatments). Ethambutol (32 μg/mL), isoniazid (8 μg/mL), streptomycin (1 μg/ mL), and rifampicin (2 μg/mL) were used as positive controls. The *Mtb* inocula was prepared from a 14-day log-phase culture, adjusted with 7H9 OADC medium to a concentration of 1.0 on the McFarland turbidity scale (OD 600 = 0.76) and subsequently diluted 1:20 with 7H9-OADC. To each well of the enzyme-linked immunosorbent assay (ELISA) plate with the above cited molecules, 100 µL of the inoculum was added, so the ﬁnal volume was 200 µL per well. The wells used as positive control, rifampicin and ethambutol were added, the negative control was the inoculum in 7H9-OADC medium, and the sterility control was non-inoculated 7H9-OADC medium. The plates were incubated for 6 days at 37ºC, after that 20 µL of the resazurin solution were added per well, followed by another incubation for 24 h at 37ºC. A change of resazurin color from blue to pink indicated its reduction to resoruﬁn, and therefore the growth of microorganisms. The minimal inhibitory concentration (MIC) in these assays was deﬁned as the lowest concentration of the molecules able to prevent the color change. All molecules’ concentrations were tested in triplicate.

*Cell culture* - The type 2 pneumocyte (T2P) cell line (A549 ATCC^®^ CCL185™ Manassas, VA, USA), was grown with RPMI-1640 (Biowest, Nuaille, FR) supplemented with 10% foetal bovine serum (FBS, Biowest, Nuaille, FR) and 100 μg/mL of antibiotic (penicillin 100X, Corning cellgro, Manassas, VA, USA). For the infection assay, the cell line was seeded in 24-well dishes plates (Corning cellgro, Manassas, VA, USA) with 1% FBS and maintained for 18 h in the presence of 5% CO_2_ at 37ºC until infection with *Mtb*.

The macrophages derived from human monocytes (MDM), were obtained according to the Declaration of Helsinki and approved by the National Committee of Ethics and National Commission of Scientific Research of the Mexican Institute of Social Security (IMSS). After obtaining written informed consent, subjects underwent venipuncture, and heparinised blood was obtained from 12 PPD-negative male healthy donors (TS-Negative subjects, aged between 18 and 35). The procedure of macrophages isolation and differentiation was carried out according to previous reports.^16^ Briefly, peripheral blood mononuclear cells (PBMCs) were isolated using Lymphosep (Biowest, Nuaille, FR). 2.5 × 10^6^ PBMC were cultured in RPMI medium using 24-well plates (Costar, Corning, NY, USA). After 2 h, non-adherent cells were removed. Subsequently, cells were differentiated for seven days with RPMI medium (Biowest, Nuaille, FR) supplemented with 10% of the decomplemented pool of human serum AB (Biowest, MO, USA). To assess macrophage phenotype, the CD68^+^ differentiation marker was evaluated using flow cytometry, obtaining a percentage of positive cells greater than 95%.

*Intracellular Mtb growth assay* - Cells were infected with *Mtb* H37Rv or MDR-MTB at a multiplicity of infection of five (MOI 5:1, bacilli: cell ratio). After 2 h of infection, cells were washed to eliminate extracellular bacteria. We determine whether ACE, N-BOC, and DFU treatments affect the ability of the cells to kill mycobacteria; thus, infected cells were treated with 2.5 to 250 µM of HDACi-based molecules during 48 h to evaluate CFU/mL in lung epithelial cells and macrophages. Then, cells were lysed with SDS 0.01% (Boehringer Mannheim, Indianapolis, USA) for 10 min, the reaction was stopped with 20% bovine serum albumin to later perform serial dilutions that were plated in triplicate onto Middlebrook 7H10 agar (DIFCO, Detroit, MI, USA), and CFU/mL were determined after 21 days of incubation at 37ºC, 5% CO_2_.

*Reverse transcription-quantitative polymerase chain reaction (RT-qPCR)* - To determine the expression of immune molecules induced by HDACi, total RNA was obtained after each infection assay using trizol reagent (Sigma, Life Science, St. Louis, USA). Reverse transcription was performed using 5 μg of total RNA, 1 μM oligoprimer (Thermo Scientific, Basingstoke, UK), 10 units of ribonuclease inhibitor (10 units/μL) (Invitrogen, Carlsbad, California), 1X RT buffer, 0.5 mM of each dNTP (Qiagen, Inc., Mexico) and four units of Omniscript reverse transcriptase (Thermo Scientific, Basingstoke, UK). Real-time qPCR was performed using a Light Cycler 480 thermocycler (Roche Applied Science Inc, USA), using the commercial reagent SSOFast (BioRad, California, USA). The primers were designed with Universal Probe Library software from Roche. The primer sequences were for HPRT (*hprt*): F-tgaccttgatttattttgcatacc- and R-cgagcaagacgttcagtcct-, LL-37 (*camp*): F-tcggatgctaacctctaccg- and R-gtctgggtccccatccat-, HBD-2 (*defb4*): F-gtctccctggaacaaaatgc- and R-gagggagccctttctgaatc-, SOD3 (*sod3*): F-aacacagtagcgccagcat- and R-ctaacagcccaggctcca-, and iNOS (*inos*): F-ttgggagttcacccagttgtg- and R-acatcgaagcggccatag-. Relative quantification of gene expression was performed by the comparative quantification cycle (Cq) method, using the formula, 2^-ΔΔCT^. This method is based on the expression levels of a target gene vs a reference gene (HPRT) comparing between treated cells. The comparative threshold cycle method was used to assess relative changes in mRNA levels between untreated cells (control) reflected in fold changes. Thus, untreated cells were uniformly normalised to a value of 1.^17^

*Immunoblotting* - iHDAC-treated cells were lysed with RIPA buffer (10 mM Tris-HCl pH 8, 1 mM EDTA, 0.5 mM EGTA, 1% Tritón x-100, 0.1% Desoxicolato, 0.1% SDS y 140 mM NaCl), then protein extract was quantified with Pierce BCA Protein Assay Kit (Thermo Fisher Scientific, USA #15045), following the manufacturer’s instructions. Cellular proteins (30 µg) were separated by sodium dodecyl sulphate-polyacrylamide gel electrophoresis (SDS-PAGE) and transferred onto nitrocellulose membranes, which were blocked with 10% non-fat milk in PBST (PBS-Triton X-100 0.5%) for 1 h at room temperature.

Monoclonal antibodies used to analyse histone acetylation were total histone H3 (rabbit Merck USA, 06-755, 1:10,000) and acetylated H3 (rabbit Merck USA, 06-599, 1:10,000). Densitometric analysis was performed using the myImageAnalysis software (Thermo Fisher Scientific), and adjusted with the loading control (β-actin, Sigma-Aldrich St.Louis USA #A5316 Mouse, 1:3000).

*Statistical analysis* - Statistical analyses were performed using the GraphPad Prism software for Mac (GraphPad Software version 6.01 San Diego California). Normal distribution was assessed using the Kolmogorov-Smirnov test for each data set, together with a non-parametric multiple comparison test of Kruskal-Wallis to identify differences among the groups. When statistical significance (p < 0.05) was found, a Dunn’s post-test was performed. Two-sided p values of < 0.05 were considered statistically significant.

*Ethics approval and consent to participate* - All studies were conducted in accordance with the Helsinki Declaration. All experimental protocols were approved by Mexican Institute of Social Security Use Committee (No-1912). Approval register R-2019-1912-075.

## RESULTS

*Selection of molecules HDACi-based* - According to Miraglia et. al.,^13^ entinostat has specific activity in HDAC1-3 isoforms, which have similar amino acid sequences in the inhibition pocket. We selected three molecules, considering the phenylenediamine aroilated functional group, availability (non-regulated drugs), and low price (Table I). Finally, we confirm HDACi activity of the selected molecules using molecular docking and compared them with the lead molecule (Table I).

**TABLE I:** Selected molecules

ZINC ID	Chemical structure/name	Binding energy (kcal/mol) *			Reported activity	Ref.
		HDAC1	HDAC2	HDAC3		
1488870	Entinostat^*^ 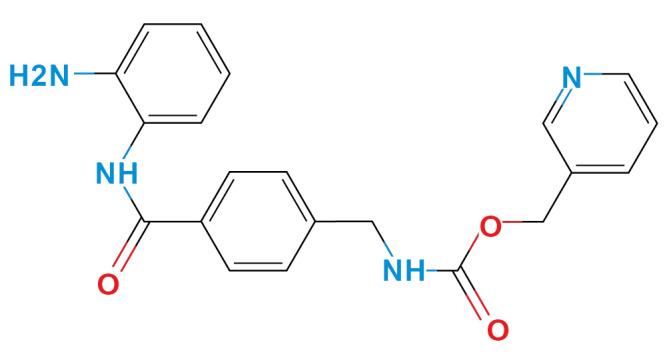	-7.6	-9.2	-8.2	Breast cancer treatment, immunomodulator	^(33,34)^
12416741	1,3-diphenylurea (DFU) 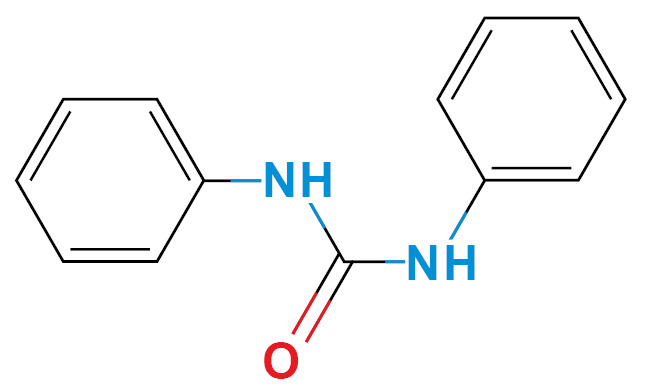	-6.3	-6.9	-6.2	Epoxide hydrolase inhibitor, K^+^ channels activator	^(35,36)^
39703	2- aminoacetanilide (ACE) 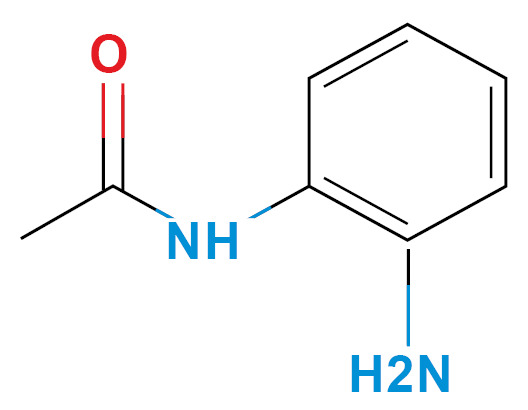	-5.3	-5.9	-5.8	HDAC candidate inhibitor, amino acetenyl derivates	^(37,38)^
39322	N-Boc-1,2- phenylenediamine (N-BOC) 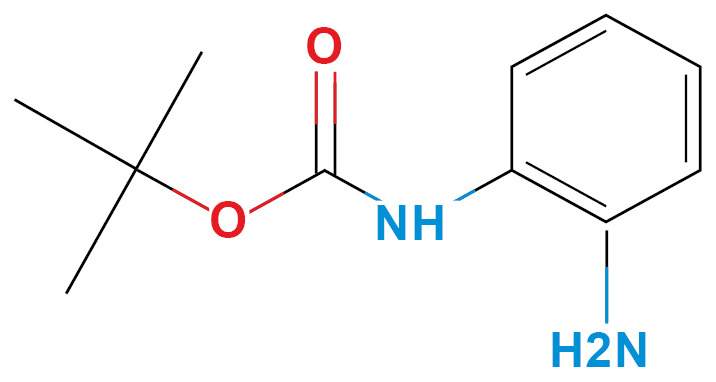	-5.3	-5.6	-5.9	HDAC candidate inhibitor, anti-inflammatory presynthetic precursor	^(39)^

*A prototypical molecule utilised as a histone deacetylase inhibitor.

*Anti-mycobacterial tuberculosis activity* - Due to the antimicrobial activity observed using butyrate in *Mtb* culture,^18^ we performed a MIC test to assess whether the three selected molecules had direct anti-mycobacterial activity. *Mtb* and MDR-MTB were grown in the presence of a serial dilution of the selected molecules or in the presence of the first line of anti-TB drugs; streptomycin, rifampicin, and isoniazid. As we expected MDR-MTB showed high resistance to isoniazid and rifampicin whereas H37Rv was inhibited at levels of 0.1 μg/mL and 1.0 μg/mL, respectively. Furthermore, the use of HDACi molecules showed that the MICs of these molecules are greater than 250 μM (Table II). Thus, subsequent results can be attributed to the activation of macrophages and epithelial cells.

**TABLE II:** Minimal inhibitory concentrations (MICs) of H37Rv and multi-drug resistance *Mycobacterium tuberculosis* (MDR-MTB)

Molecule	H37Rv	MDR-MTB
Streptomycin (S)	0.8 µg/mL	0.8 µg/mL
Rifampicin (R)	1.0 µg/mL	> 1.0 µg/mL
Isoniazid (H)	0.1 µg/mL	> 0.1 µg/mL
1,3-Diphenylurea	> 250 μM	> 250 μM
aminoacetanilide	> 250 μM	> 250 μM
N-Boc-1,2-phenylenediamine	> 250 μM	> 250 μM

**Fig. 1:**
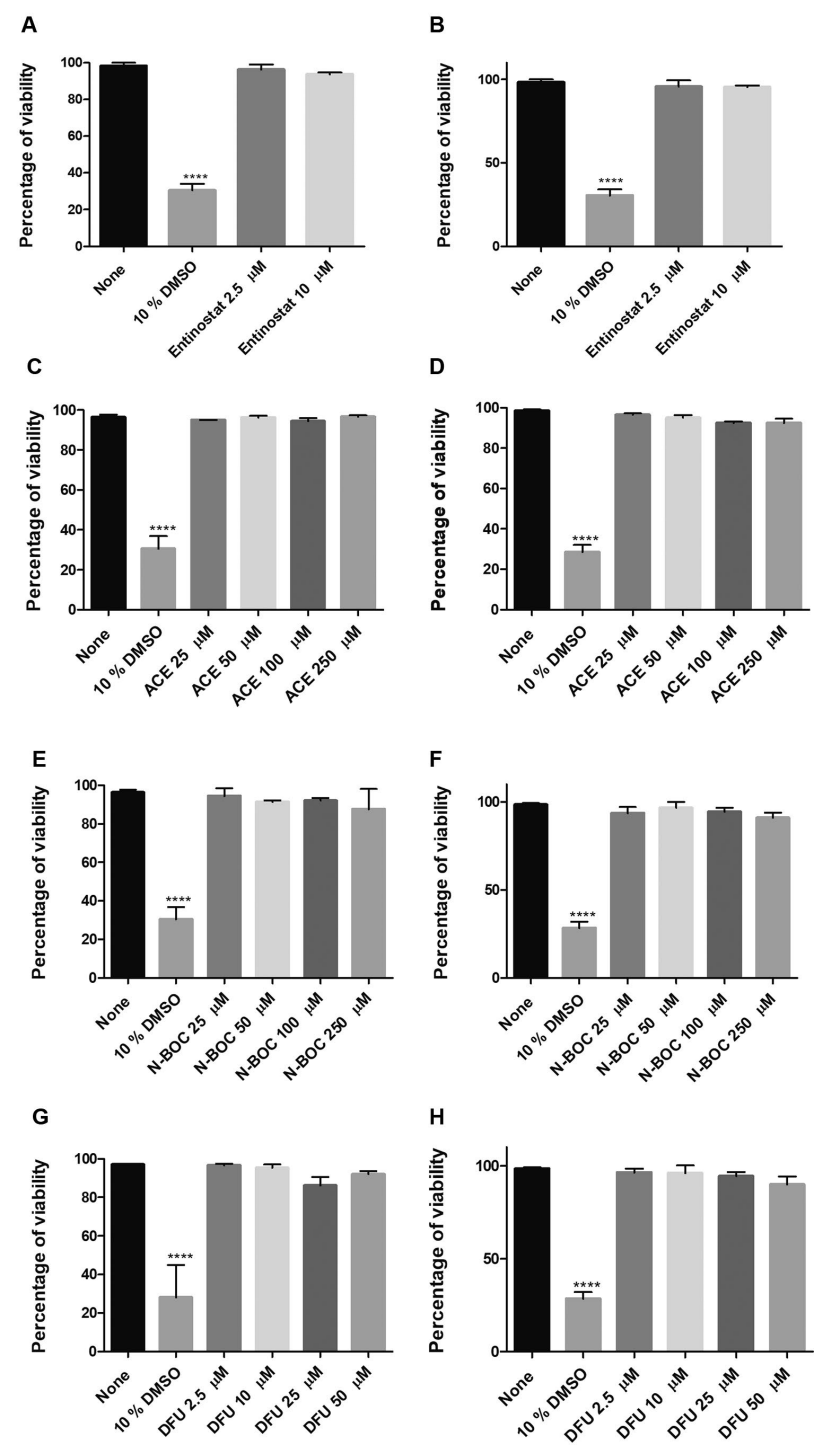
viability of the cells treated with histone deacetylases inhibitors (HDACi). T2P (A, C, E, G) and macrophages derived from human monocytes (MDM) (B, D, F, H) were cultured in the presence of various concentrations of entinostat, aminoacetanilide (ACE), N-Boc-1,2-phenylenediamine (N-Boc) and 1,3-Diphenylurea (DFU) for 48 h. Cell viability was determined using the Guava^®^ ViaCount™ assay. Data are expressed as mean ± standard deviation (SD). Statistics were calculated by Kruskal-Wallis and Dunn’s post-hoc tests. In each experimental group, n = 3. **** p < 0.0001.

*HDACi molecules reduce CFU counts in macrophages and type II pneumocytes* - To investigate the toxic effect of HDACi-selected molecules on epithelial cells and MDM, we first evaluated a range of concentrations based on entinostat activity (2.5 µM to 250 µM)(Fig. 1A-B).^12^ The results showed that treatment with ACE and N-Boc at concentrations of 250 µM does not affect cell viability (Fig. 1C-F). The use of concentrations greater than 50 µM of DFU significantly affects the viability of epithelial cells. Thus, we evaluated only concentrations lower than 50 µM (Fig. 1G-H).

**Fig. 2:**
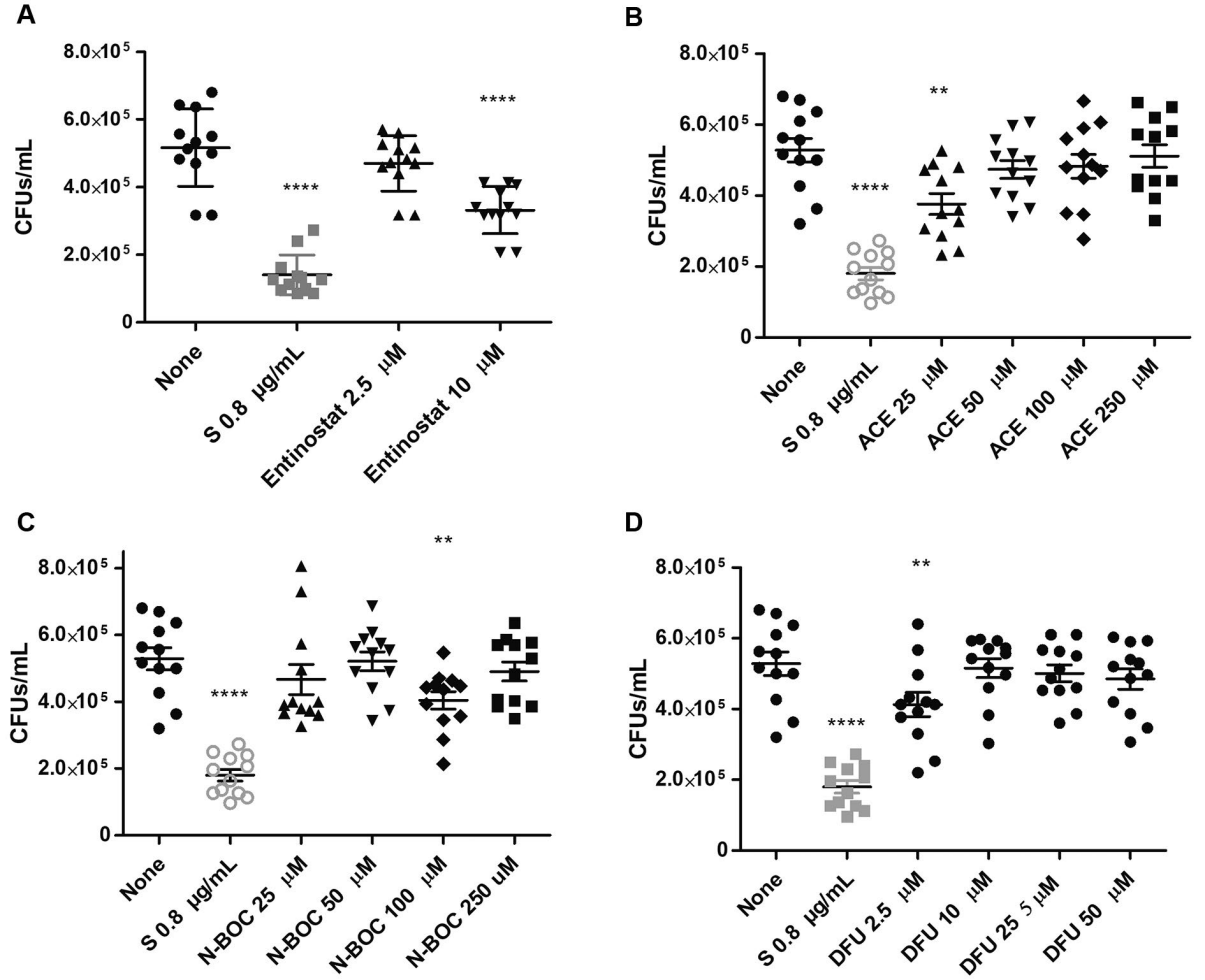
histone deacetylases inhibitors (HDACi)-based molecules reduce colony-forming unit (CFU) in macrophages derived from human monocytes (MDM). Cells were infected with *Mycobacterium tuberculosis (Mtb)* with a MOI of 5 and treated with entinostat (A) aminoacetanilide (ACE) (B), N-Boc-1,2-phenylenediamine (N-Boc) (C), 1,3-Diphenylurea (DFU) (D) and streptomycin (S) for 48 h. Afterwards, cells were lysed, and then it was determined the CFUs by serial dilutions and macrophages. Data are expressed as mean ± standard deviation (SD). Statistics were calculated by Kruskal-Wallis and Dunn’s post-hoc tests. In each experimental group, n = 12 *p < 0.05, **p < 0.01, *** p < 0.001, **** p < 0.0001.

**Fig. 3:**
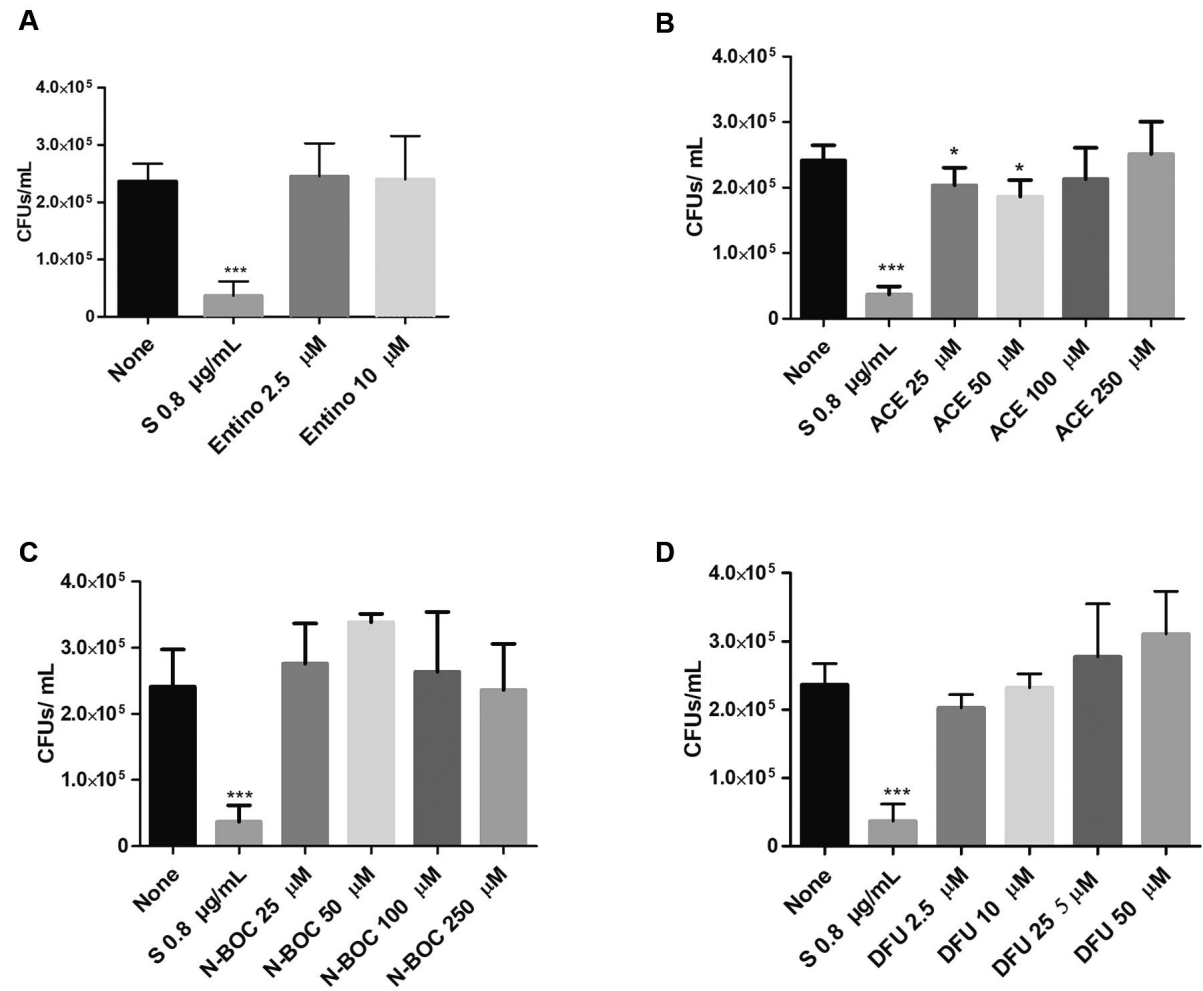
histone deacetylases inhibitors (HDACi)-based molecules reduce colony-forming unit (CFU) in type 2 pneumocytes. Cells were infected with *Mycobacterium tuberculosi*s (*Mtb*) with a MOI of 5 and treated with entinostat (A), aminoacetanilide (ACE) (B), N-Boc-1,2-phenylenediamine (N-Boc) (C), 1,3-Diphenylurea (DFU) (D) and streptomycin (S) for 48 h. Afterwards, cells were lysed, and then it was determined the CFUs by serial dilutions and macrophages. Data are expressed as mean ± standard deviation (SD). Statistics were calculated by Kruskal-Wallis and Dunn’s post-hoc tests. In each experimental group, n = 12 *p < 0.05, **p < 0.01, *** p < 0.001, **** p < 0.0001.

Next, we evaluated whether the selected molecules decreased the bacterial load of *Mtb*-infected macrophages. Results showed that MDM treated with entinostat 10 µM (Fig. 2A), showed a significant reduction regarding the total H37Rv burden compared to untreated conditions. Similarly, we observed that ACE decreased H37Rv growth at 25 µM (Fig. 2B), while N-BOC and DFU decreased mycobacterial growth at 100 µM (Fig. 2C) and 2.5 µM (Fig. 2D) respectively. Whereas ACE 25 and 50 µM promote clearance in T2P (Fig. 3B). In summary, we proved that N-BOC and DFU increased the *Mtb* macrophage-mediated killing, and ACE is active in both cells type.

**Fig. 4:**
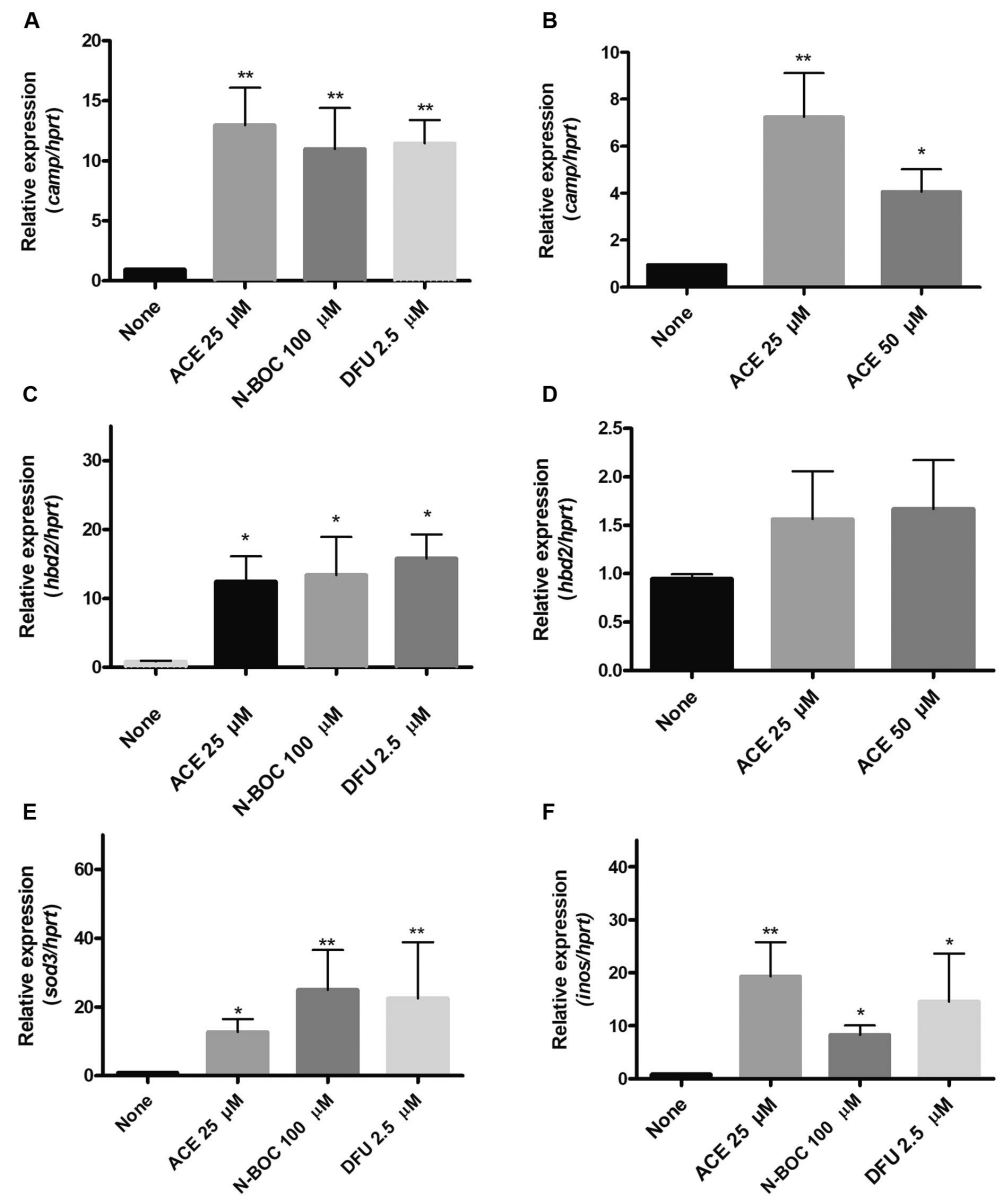
histone deacetylases inhibitors (HDACi) induce antimicrobial molecules to promote mycobacterium killing. To evaluate whether aminoacetanilide (ACE), N-Boc-1,2-phenylenediamine (N-Boc), 1,3-Diphenylurea (DFU) induced the expression of CAMP and DEFB4 in macrophages (A, C) and type 2 Pneumocites (B, D) both genetic expressions were valued. Furthermore, we valued the induction of superoxide dismutase (SOD)3, and iNOS in macrophages infected (E, F). Data are expressed as mean ± standard deviation (SD). Statistics were calculated by Kruskal-Wallis and Dunn’s post-hoc tests. In each experimental group, n = 6. * p<0.05, **p < 0.01.

*The treatment with HDACi-based molecules is a potent inductor of antimicrobial peptides and reactive oxygen and nitrogen species* - Several reports have described that entinostat increases the expression of AMPs.^10^ Therefore, we evaluated whether ACE, N-BOC, and DFU up-regulate the expression of LL-37 and HBD-2, leading to the elimination of *Mtb*. First, we evaluated LL-37 gene expression (*camp*) in MDMs and T2P. Previous reports by our group demonstrated that *Mtb* induced LL-37 expression 3-4 times more than controls without infection.^19^ Interestingly, as shown in Fig. 4A, all treatments in MDMs induced significant changes in CAMP expression. In T2P, we observed only a higher CAMP expression induced by ACE 25 µM (Fig. 4B).

Thereafter, we analysed the gene expression of HBD-2 (*defb4*). Our results showed that in MDM, *defb4* is upregulated by ACE, N-BOC, and DFU (Fig. 4C). In T2P none of the treatments induced significant changes in the *defb4* expression (Fig. 4D).

Oxidative and nitrosative stress are key factors to eliminate *Mtb* during infection.^20^ Thus, to explore whether HDACi modifies the expression levels of superoxide dismutase (SOD)-3 and inducible nitric oxide synthase (iNOS), we evaluated their expression after 18 h of *Mtb* infection. The use of ACE, N-BOC, and DFU in MDM infected increase gene expression of both SOD and iNOS (Fig. 4E-F). These results showed that the selected molecules promoted important microbicidal mechanisms to control of tuberculosis infection.

**Fig. 5:**
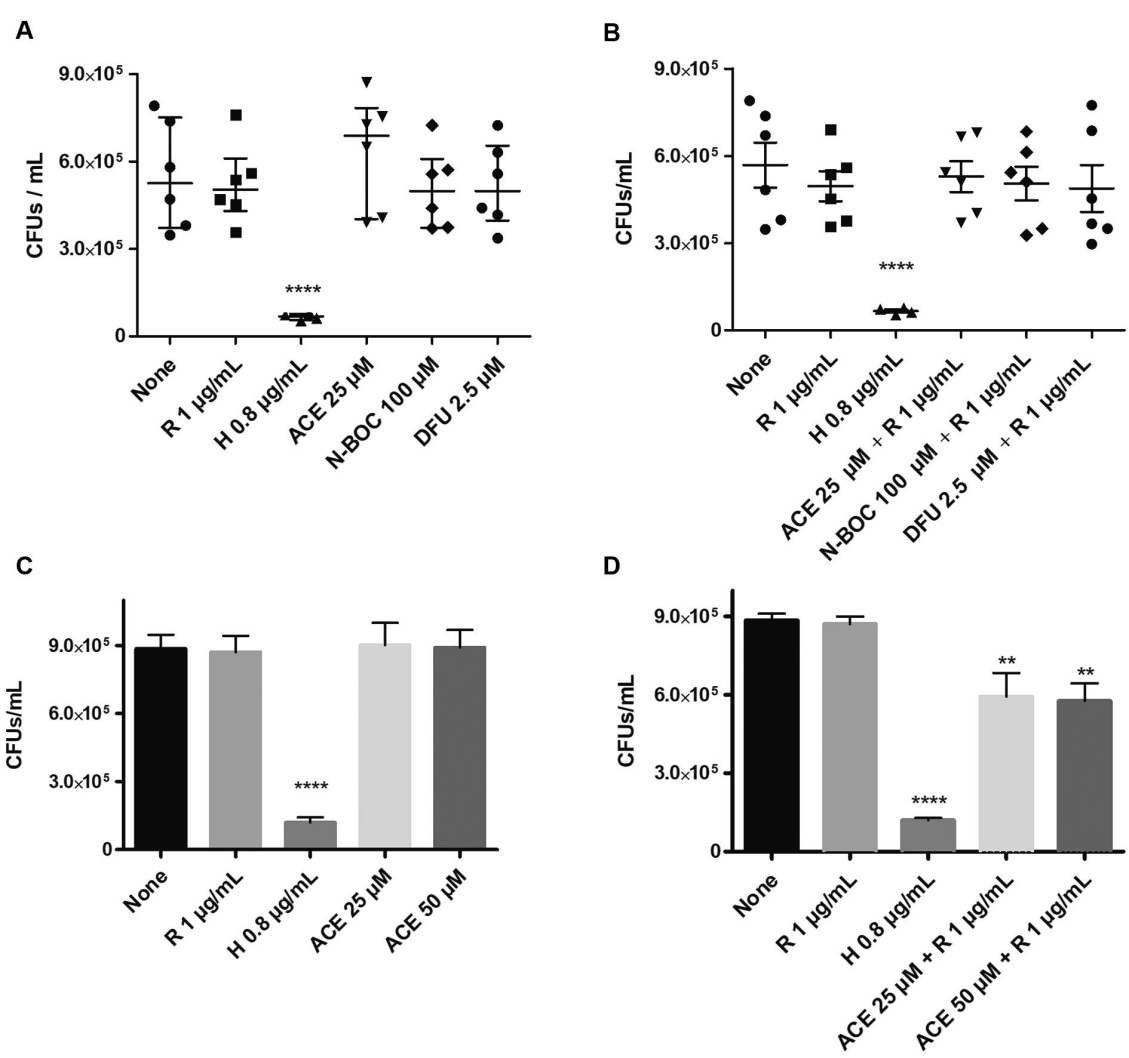
treatment of histone deacetylases inhibitors (HDACi)-based molecules with rifampicin reduce colony-forming unit (CFU)/mL of multi-drug resistance *Mycobacterium tuberculosis* (MDR-MTB) in type 2 pneumocytes. T2P were infected with *Mtb* at a MOI of 5 and treated with Streptomycin (S), aminoacetanilide (ACE), N-Boc-1,2-phenylenediamine (N-Boc), 1,3-Diphenylurea (DFU) alone or in combination with rifampicin (R) at 48 h. Isoniazid (H) was used as control. Subsequently, cells were lysed and CFU were evaluated. Graphs show mean ± standard deviation (SD) from six independent experiments by duplicate. Statistics were calculated by Kruskal-Wallis and Dunn’s post-hoc tests. *p < 0.05; **p < 0.01, ***p < 0.001, ****p<0.0001.

*ACE in combination with rifampicin promotes the drug sensibility in MDR strains* - To assess whether the AMPs upregulation decreased the burden of MDR-MTB, we evaluated CFU counts from infected cells treated with the selected molecules alone or in combination with conventional anti-TB drugs (Fig. 5A-B). None of the treatments showed significant effects, however, when we evaluated them in combination with rifampicin in MDR-MTB infected cells, the ACE decreased in both concentrations the *Mtb* intracellular growth in T2P (Fig. 5C-D).

**Fig. 6:**
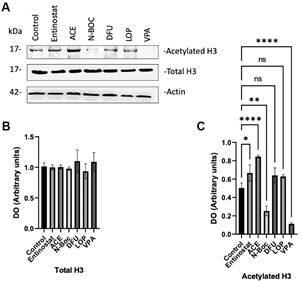
analysis of H3 acetylation in pneumocytes type II under histone deacetylases inhibitors (HDACi) candidates’ treatment. Pneumocytes type II were treated with 10 µM Entinostat, 2.5 µM 1,3- Diphenylurea (DFU), 25 µM aminoacetanilide (ACE) 100 µM N-Boc-1,2-phenylenediamine (N-Boc), 3 mM Loperamide (LOP) and 1 mM Valproic acid (VPA). (A) Western blot analysis on total H3 and H3 acetylation levels, (B) The graph represents total histone 3 (H3) levels and (C) Acetylated H3 compared to β-actin. Levels were analysed using a one-way analysis of variance (ANOVA) with Tukey’s post-hoc analysis. The values are expressed as means ± standard error of the mean (SEM) of three independent experiments. **p < 0.001, ****p < 0.0001.

*Predicted in silico molecules promote histone acetylation* - To determine whether the examined molecules exhibited iHDAC activity, we conducted a western blot analysis (Fig. 6A). Our findings revealed that cells treated with entinostat, DFU, ACE, N-BOC, LOP and VPA did not alter H3 expression (Fig. 6B). However, these compounds induced histone acetylation, except for N-BOC and VPA (Fig. 6C) In addition, we evaluated a nonselective iHDAC, valproic acid (VPA) at 1 mM, and a non-iHDAC molecule, loperamide (LOP), at 3 mM.

## DISCUSSION

In the past few years, the development and use of compounds that boost the host immune response are promising treatment strategies for the future of multidrug-resistant TB therapy.^21^ HDACi is known to increase immune response through gene transcription promotion and inflammation regulation in infectious diseases.^6^ In this study, we repositioned and evaluated three molecules based on entinostat to promote antimicrobial activity against *Mtb*, increasing antimicrobial peptides expression and respiratory burst on MDM and T2P.

First, we selected three molecules based on their ability to bind catalytic sites of HDAC 1-3 isoforms by the presence of the phenylenediamine functional group. Thus, we used entinostat as a lead molecule.^12^ The molecular docking shows substantial differences; ACE and N-BOC have selectivity to the HDAC 2 and 3, while DFU to isoforms 1 and 2. Besides, only N-BOC did not bind to zinc. The presence of the tert-Butyl functional group in N-BOC could give steric impediment preventing its entry into the inhibition pocket, showing only the capping capacity to the HDAC 1-3 (data no shown).

Several *in vitro* studies have shown that class I HDAC inhibitors enhance antimicrobial peptide expression and promote intracellular bacterial killing in macrophages.^22^ ^,^ ^23^ Consistent with the above described, in our study, we observed that *Mtb* survival decreases in macrophages treated with entinostat 10 µM, ACE 25 µM, N-BOC 100 µM, and DFU 2.5 µM. Nonetheless, the antimicrobial activity of T2P increased only with ACE 25 and 50 µM, and entinostat did not show activity in these cells. Thus, we suggest that HDACi-based molecules have a potential specific microbicidal effect depending on the cell type to promote the immune response and bacterial control, thus the use of these molecules for other tissue infections must be evaluated according each cell type.

The antimicrobial peptides, including β-defensins and LL-37, are essential molecules to control and eliminate intracellular *Mtb*.^10^ Indeed, the susceptibility to develop tuberculosis correlates with a low plasmatic concentration of AMPs.^24^ Thus, we next measured the expression of LL-37 and HBD-2 in infected cells treated with ACE, N-BOC, and DFU. We found that all tested HDACi increased LL-37 expression in the studied cells. Therefore, we suggest that the phenylenediamine functional group, present in ACE, N-BOC, and DFU, activates pathways related to antimicrobial activity. Indeed, previous reports have shown that entinostat allows STAT3 and HIF1α acetylation that subsequently induces LL-37 up-regulation.^11^ In contrast, HBD-2, a potent chemotactic peptide with high antibacterial activity, increased in MDMs with all treatments but not in T2P. Although ACE does not increase HBD-2 expression in T2P, it promotes mycobacterial clearance. In a previous report, HDACi activates the induction several antimicrobial peptides, such as HBD-1 in lung epithelial cell lines.^25^ Therefore, we hypothesise that other antimicrobial peptides might be up regulated by the evaluated molecules, but more studies are needed to further elucidate this issue. Besides, in other studies, HDACi promoted P65 acetylation and NF-kB activation, which is a key transcription factor during the induction of AMPs and inflammatory response.^26^

Induction of reactive oxygen species by SOD3 involved during the inflammatory response promotes *Mtb* control.^27^ Furthermore, iNOS, the enzymatic source of Nitric Oxide (NO), is an immune response mediator in macrophages.^28^ Thus, we evaluated SOD3 and iNOS gene expression. Our observations demonstrated that ACE, N-BOC, and DFU promoted SOD3 and iNOS expression in macrophages infected with *Mtb*. Also, in other reports, valproic acid induced iNOS and high levels of NO in macrophages infected with *Mtb*.^29^ Thus, this is the first report on SOD3 induction by HDACi treatment during *Mtb* infection, similar results have been reported elsewhere in *Salmonella typhimurium* or *Escherichia coli* infection.^30^ Overall, our results suggest that the molecules used throughout the present study, promote *Mtb* killing also by respiratory bursts in macrophages.

Interestingly, we observed that the combination of rifampicin and ACE decreased the MRD-MTB load in T2P, these cells have been referred as active replicating niche for *Mtb*. Rifampicin modulates signaling mechanisms involved in autophagy.^31^ In addition, as we described above, LL-37 is up-regulated by ACE. Thus, the proposed mechanism is that ACE enhances autophagy through LL-37 induction, improving the antimicrobial effect. Similar studies reported that the combination of vitamin D, sodium butyrate (HDACi) and isoniazid in MDR-MTB infected cells, increased autophagy in infected cells leading to the control of *Mtb* growth.^32^ Certainly, one handicap for the present study is that we did not evaluated the induction of autophagy by the studied compounds.

*In conclusion* - In summary, the selection of HDACi-based molecules provides a novel strategy to control *Mtb*, We experimentally demonstrated that the studied molecules promoted histone acetlytaion which led to the expression of antimicrobial peptides, SOD3, and INOS expression. Furthermore, we found that the combination of ACE and rifampicin increases the susceptibility of MDR-MTB to rifampicin. These data support the potential use of HDACi in TB therapy.
